# Antagonist anti-LIF antibody derived from naive human scFv phage library inhibited tumor growth in mice

**DOI:** 10.1186/s12865-024-00636-w

**Published:** 2024-08-22

**Authors:** Shengyan Zhao, Han Deng, Ying Lu, Yiran Tao, David Li, Xiaohua Jiang, Xian Wei, Xiaofeng Chen, Fanxin Ma, Yuxi Wang, Lantu Gou, Jinliang Yang

**Affiliations:** 1 Department of Biotherapy, Cancer Center, State Key Laboratory of Biotherapy/Collaborative Innovation Center for Biotherapy, West China Hospital, Sichuan University, Chengdu, 610041 China; 2 Department of Pulmonary and Critical Care Medicine, Targeted Tracer Research and Development Laboratory, Institute of Respiratory Health, Frontiers Science Center for Disease-related Molecular Network, National Clinical Research Center for Geriatrics, State Key Laboratory of Biotherapy, Precision Medicine Key Laboratory of Sichuan Province & Precision Medicine Research Center, West China Hospital, Sichuan University, Chengdu, 610041 Sichuan China; 3 West China-California Research Center for Predictive Intervention Medicine, West China Hospital, Sichuan University, Chengdu, 610041 Sichuan China; 4 503 Central Avenue, Sunnyvale, 94086 California United States of America; 5 Sound Biopharmaceuticals Co., Ltd, Tianfu International Bio-Town, Huigu Dong 2nd Road 8, 610200 Chengdu, Sichuan China; 6 Research Unit of Gene and Immunotherapy, Chinese Academy of Medical Sciences, Chengdu, China

**Keywords:** LIF, LIFR, gp130, STAT3, Antagonist antibodies, Tumor therapy

## Abstract

**Background:**

Leukemia inhibitory factor (LIF) is a multifunctional member of the IL-6 cytokine family that activates downstream signaling pathways by binding to the heterodimer consisting of LIFR and gp130 on the cell surface. Previous research has shown that LIF is highly expressed in various tumor tissues (e.g. pancreatic cancer, breast cancer, prostate cancer, and colorectal cancer) and promotes cancer cell proliferation, migration, invasion, and differentiation. Moreover, the overexpression of LIF correlates with poor clinicopathological characteristics. Therefore, we hypothesized that LIF could be a promising target for the treatment of cancer. In this work, we developed the antagonist antibody 1G11 against LIF and investigated its anti-tumor mechanism and its therapeutic efficacy in mouse models.

**Results:**

A series of single-chain variable fragments (scFvs) targeting LIF were screened from a naive human scFv phage library. These scFvs were reconstructed in complete IgG form and produced by the mammalian transient expression system. Among the antibodies, 1G11 exhibited the excellent binding activity to human, cynomolgus monkey and mouse LIF. Functional analysis demonstrated 1G11 could block LIF binding to LIFR and inhibit the intracellular STAT3 phosphorylation signal. Interestingly, 1G11 did not block LIF binding to gp130, another LIF receptor that is involved in forming the receptor complex together with LIFR. In vivo, intraperitoneal administration of 1G11 inhibited tumor growth in CT26 and MC38 models of colorectal cancer. IHC analysis demonstrated that p-STAT3 and Ki67 were decreased in tumor tissue, while c-caspase 3 was increased. Furthermore, 1G11 treatment improves CD3+, CD4 + and CD8 + T cell infiltration in tumor tissue.

**Conclusions:**

We developed antagonist antibodies targeting LIF/LIFR signaling pathway from a naive human scFv phage library. Antagonist anti-LIF antibody exerts antitumor effects by specifically reducing p-STAT3. Further studies revealed that anti-LIF antibody 1G11 increased immune cell infiltration in tumor tissues.

**Supplementary Information:**

The online version contains supplementary material available at 10.1186/s12865-024-00636-w.

## Introduction

Leukemia inhibitory factor (LIF) is a multifunctional cytokine. It is a member of the interleukin-6 (IL-6) family [1, 2]. LIF binds to the heterodimer composed of LIFR and gp130 to promote the phosphorylation of the Janus kinase (JAK) receptor, thereby initiating intracellular signal transduction [3, 4]. LIF plays pivotal roles in various cells, tissues and organs, such as regulating pregnancy, promoting self-renewal of pluripotent stem cells, and influencing differentiation and regeneration of the nervous system [5–7].

Previous studies have shown that the expression of LIF is increased in various tumor types, including breast cancer [8], pancreatic cancer [9], nasopharyngeal cancer [10], and prostate cancer [11]. Through autocrine and paracrine means, LIF can selectively activate different signaling pathways in cells, such as PI3K/AKT and JAK/STAT3 [12]. In pancreatic ductal adenocarcinoma (PDAC), KRAS mutations upregulate LIF through the MEK/ERK cascade, thereby promoting cancer malignancy [13]. Moreover, LIF produced by pancreatic stellate cells (PSCs) in PDAC activates STAT3 and is involved in the regulation of cancer cell differentiation and epithelial-mesenchymal transition [14]. Furthermore, the LIFR-Hippo-YAP pathway has been demonstrated to facilitate the proliferation, migration, and invasion of gastric cancer cells, while simultaneously inhibiting apoptosis [15]. The critical roles of LIF in tumorigenesis and tumor progression have been highlighted. Therefore, developing monoclonal antibodies (mAbs) targeting LIF to disrupt the downstream signaling pathway may be a potential strategy for cancer treatment.

Here, we generated the anti-LIF monoclonal antibody 1G11 with the ability to cross-recognize human, monkey, and mouse LIF from a naive human scFv phage library. 1G11 specifically blocks LIF binding to LIFR without affecting LIF binding to gp130 and inhibits the LIF/LIFR/STAT3 signaling pathway in vitro and in vivo. In addition, 1G11 treatment effectively suppressed tumor cell proliferation and promoted apoptosis, while also enhancing T-cell infiltration. These effects ultimately inhibited tumor growth in mice. Our results indicate that 1G11 has potential as an antitumor drug.

## Materials and methods

### Cell lines

Human multiple myeloma cell line (U266), human colon carcinoma cell line (HCT116), mouse colon cancer cell lines (CT26 and MC38), and human embryonic kidney cell line 293 F (HEK293F) were stored in our laboratory. The cell lines were cultured in a humidified incubator at 37℃ with 5% CO_2_ in standard cell culture media according to the manufacturer’s specifications.

### Construction and biopanning of the naive human scFv phage library

The construction of the naive human scFv phage library was performed according to published protocols with minor modifications [16]. The phage library was biopanned with streptavidin magnetic beads against biotinylated hLIF. Detailed methods of this process have been described in earlier studies [17].

### ELISA

The microtiter plate wells were coated with LIF (50 ng/well) at 37 °C for 2 h. After the plate was blocked with 3% bovine serum albumin (BSA), the phage supernatants or recombinant antibodies were added and incubated at 37 °C for 1 h. The plates were washed with PBST and incubated with HRP-conjugated mouse anti-M13 antibody or HRP-conjugated goat anti-mouse IgG antibody. Finally, TMB was added and the reaction was stopped by 2 M H_2_SO_4_. The absorbance was measured at 450 nm.

For receptor competitive ELISA, the microtiter plate wells were coated with LIFR-his or gp130-his (100 ng/well). Recombinant antibodies were mixed with hLIF-hFc (50 ng) and incubated at 4℃ for 1 h. The mixture was added to the BSA-blocked plate and incubated at 37℃ for 1 h. The plates were washed with PBST and incubated with HRP-conjugated goat anti-human IgG antibody. TMB and H_2_SO_4_ were then added to the plate. The absorbance was measured at 450 nm.

### Kinetics of the binding of phage supernatant to LIF

BLI assays were performed using the Gator Label-Free Bioanalysis (Gator Bio, US). The Protein A probes were prewetted in k buffer for 300 s. The measurement of the sample probe began with 60 s of baseline measurement in k buffer, followed by 120 s of loading with 10 µg/mL LIF-hFc. The probe was washed in the k buffer for 60 s and incubated with the phage supernatant for 180 s, followed by incubation in the k buffer for 180 s. The reference probe measurement was the same as the sample probe measurement, but no LIF-hFc load was applied.

### Recombinant protein expression

The heavy chain and light chain of the selected anti-LIF scFvs in the phage were sequenced, synthesized and inserted into mammalian expression vectors (pTT5-mk and pTT5-mIgG2a, General Biosystems, China). HEK293F cell suspension (2 × 10^6^ cells/mL, 90 mL) was prepared the day before transfection. 100 µg of recombinant antibody plasmids were diluted into 5 mL of the expression medium, with a 1:1 ratio of heavy chain to light chain. Next, 325 µg linear PEI (MW 25,000, Polyscience, USA) was diluted into 5 mL of the expression medium. The PEI mixture was then mixed with the plasmid mixture, incubated at room temperature for 15 min, and added to the cell suspension. After 6 days, the cell culture supernatant was harvested and purified by protein G affinity chromatography. Human and mouse LIF with human IgG1-Fc tag (hLIF-hFc, mLIF-hFc) were expressed and purified in a manner consistent with recombinant antibodies.

The other recombinant protein sources were as follows: human LIF (Z02681, Genscript, China), biotinylated human LIF (LIF-H82E2, Acrobiosystems, China), mouse LIF (250-02, PeproTech, USA), cynomolgus LIF (RP1074Y-100, Kingfisher Biotech, USA), human LIFR-his (10,628-H08H, Sino Biological, China), and human gp130-his (10,974-HCCH, Sino Biological, China).

### Surface plasmon resonance (SPR)

Biacore 8 K system (Cytiva, USA) was used for SPR analysis. All samples flowing through the chip were diluted with running buffer (pH 7.4) containing 3 mM EDTA, 10 mM HEPES, 150 mM NaCl, and 0.05% Tween-20 at 25℃. Anti-mouse IgG antibody was immobilized on the CM5 chip by amine-coupling. 1G11 was diluted in running buffer and captured on the chip surface. For affinity analysis, different concentrations of LIF were run through the control and detection channels. For receptor competition analysis, 200 nM of hLIF flowed on the chip followed by 500 nM of LIFR-his or gp130-his. Finally, 10 mM glycine-HCl solution (pH 1.5) was used to regenerate the chip.

### PhosphoSTAT3 determination

1 × 10^6^ cells ( U266 or HCT116 ) were plated on the plates (2 mL/well) and cultured overnight. 50 µL hLIF (1 µg/mL) was incubated with equal volumes of antibodies for 1 h. The hLIF/antibody mixture was then added to plates and cultured at 37℃ for 20 min. Cells were lysed for protein extraction with RIPA lysis buffer containing protease and phosphatase inhibitors. Western-blot was used to quantify STAT3 phosphorylation (Tyr705).

### Antitumor activity experiment in vivo

Female BALB/c mice and C57BL/6 mice (6 weeks old) were purchased from Vital River Laboratory (Beijing, China). 5 × 10^5^ CT26 or MC38 cells were injected subcutaneously into the right side of BALB/c or C57BL/6 mice, respectively. 1G11 and isotype control mouse IgG were intraperitoneally injected every three days for four injections at the dose of 10 mg/kg when the tumor volume reached approximately 100 mm^3^. Tumor volume was calculated using the formula: V = (long axis) × (short axis)^2^ × 0.5. To calculate tumor growth inhibition (TGI, in%), we divided the tumor volumes in the treatment groups by those in the control groups. Mice were anesthetized using isoflurane and then sacrificed by cervical dislocation on day 15, and peripheral blood, tumor tissues, and major organs were collected for further analysis.

### FACS analysis

RPMI-1640 medium containing 1 mg/mL collagenase type IV (Thermo Fisher Scientific, Waltham, US) and 0.5 mg/mL DNaseI (Thermo Fisher Scientific, Waltham, US) was used to digest tumor tissues isolated from CT26 cell tumor-bearing mice for 1.5 h at 37℃. Tumor cells were passed through a 70 μm cell strainer. A CD16/32 blocker (BD Biosciences, California, US) was used to block the single cell suspensions after three washings with PBS containing 5% FBS. Fixable Viability Stain 575 V (BD Biosciences, California, US) was used to label the single cells, followed by staining with anti-mouse antibodies for 30 min at 4℃: CD45-APC-Cy7, CD3-BB700, CD8-PE-Cy7, and CD4-APC. These antibodies were purchased from BD Biosciences (California, USA).

### Immunohistochemical (IHC) staining

Formalin-fixed paraffin-embedded tissue sections were processed to remove paraffin and then subjected to immunohistochemical analysis using specific antibodies. p-STAT3 (Tyr705) antibody was purchased from Abcam (Cambridge, UK). Cell proliferation marker Ki-67 and apoptotic marker c-caspase 3 (CC3) antibodies were obtained from Servicebio (Wuhan, China).

### Statistical analysis

Statistical analysis was performed using GraphPad Prism 8.0. The results are expressed as mean ± standard deviation (SD). Two-way ANOVA and t-test were utilized for the statistical analysis of the data where appropriate. For the purpose of rejecting the null hypothesis and determining the statistical significance of the data, *P* > 0.05 was used as the threshold. For the statistical significance, the figures show the following standard abbreviations: ∗(*P* < 0.05), ∗∗(*P* < 0.01), ∗∗∗(*P* < 0.001), ∗∗∗∗(*P* < 0.0001), and ns (no statistical significance, *P* ≥ 0.05).

## Results

### Construction of scFv phage library and screening of anti-LIF scFv

**Fig. 1:**
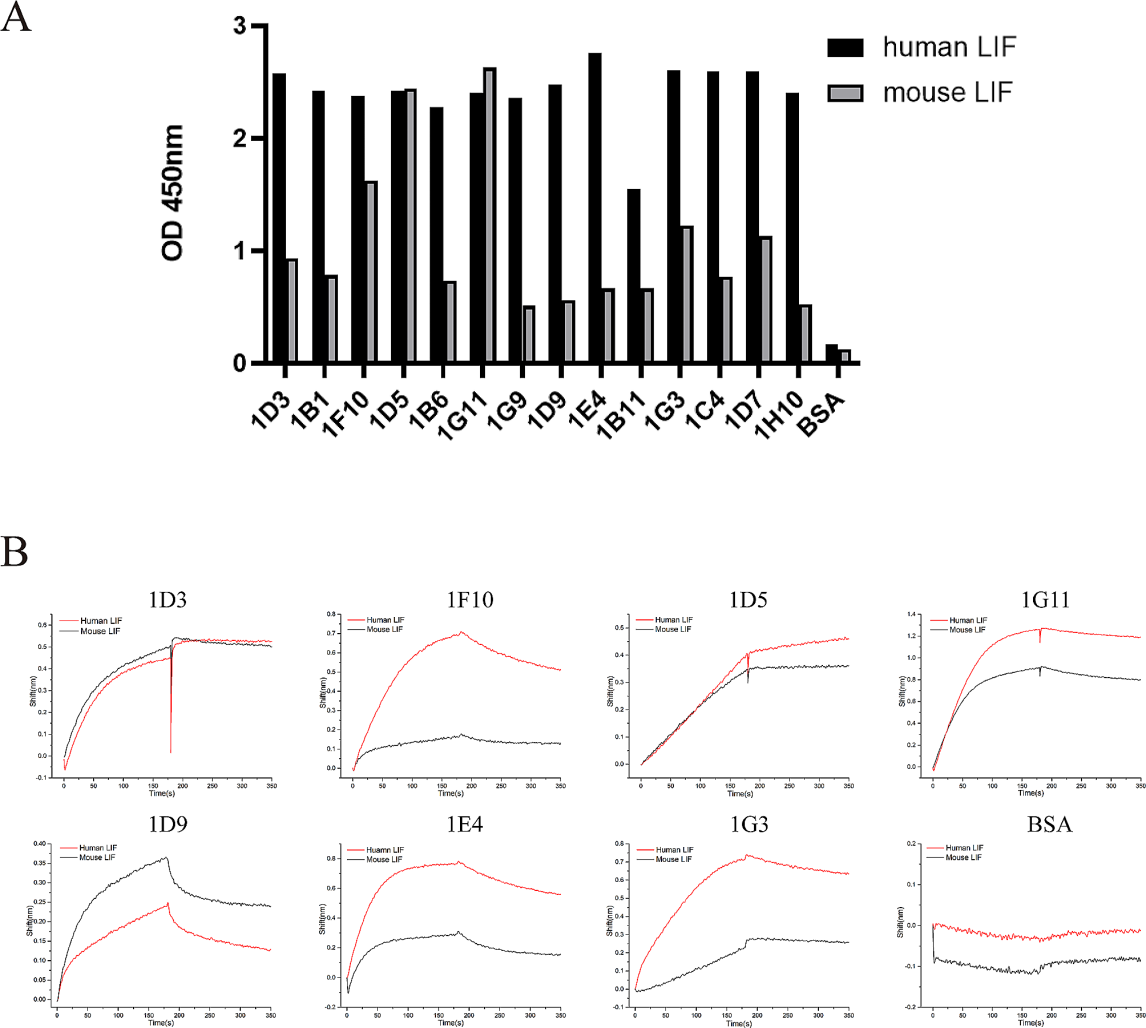
Screening of anti-LIF scFvs by phage display. (**A**) Identification of the binding selectivity of 14 clones by phage ELISA. Phage clones binding to human LIF (black bars) and mouse LIF (gray bars) were detected by the HRP-conjugated anti-M13 phage antibody. (**B**) Binding kinetics of 7 phage supernatants with human LIF (red) and mouse LIF (black) were measured by Gator. BSA was used as a negative control

In this study, a naive human scFv phage library was constructed. Biotinylated hLIF was incubated with the scFv phage library in the liquid phase, and the phages bound to biotinylated hLIF were screened with streptavidin magnetic beads. Through two rounds of biopanning, 96 phage clones were then randomly selected for phage ELISA analysis. There were 14 clones that recognized both hLIF and mLIF (Fig. 1A). The Gator Label-Free bioassay system enables direct analysis of the kinetics of scFvs on the phage surface, eliminating the requirement for antibody expression. Thus, the kinetics of these clones with hLIF and mLIF were measured using Gator. Next, 7 clones exhibited superior dynamic characteristics with hLIF and mLIF (Fig. 1B). Then 7 positive clones were selected for variable region sequencing and the sequences were analyzed using NCBI-BLAST.

### Recombinant antibody expression and activity assay

**Fig. 2:**
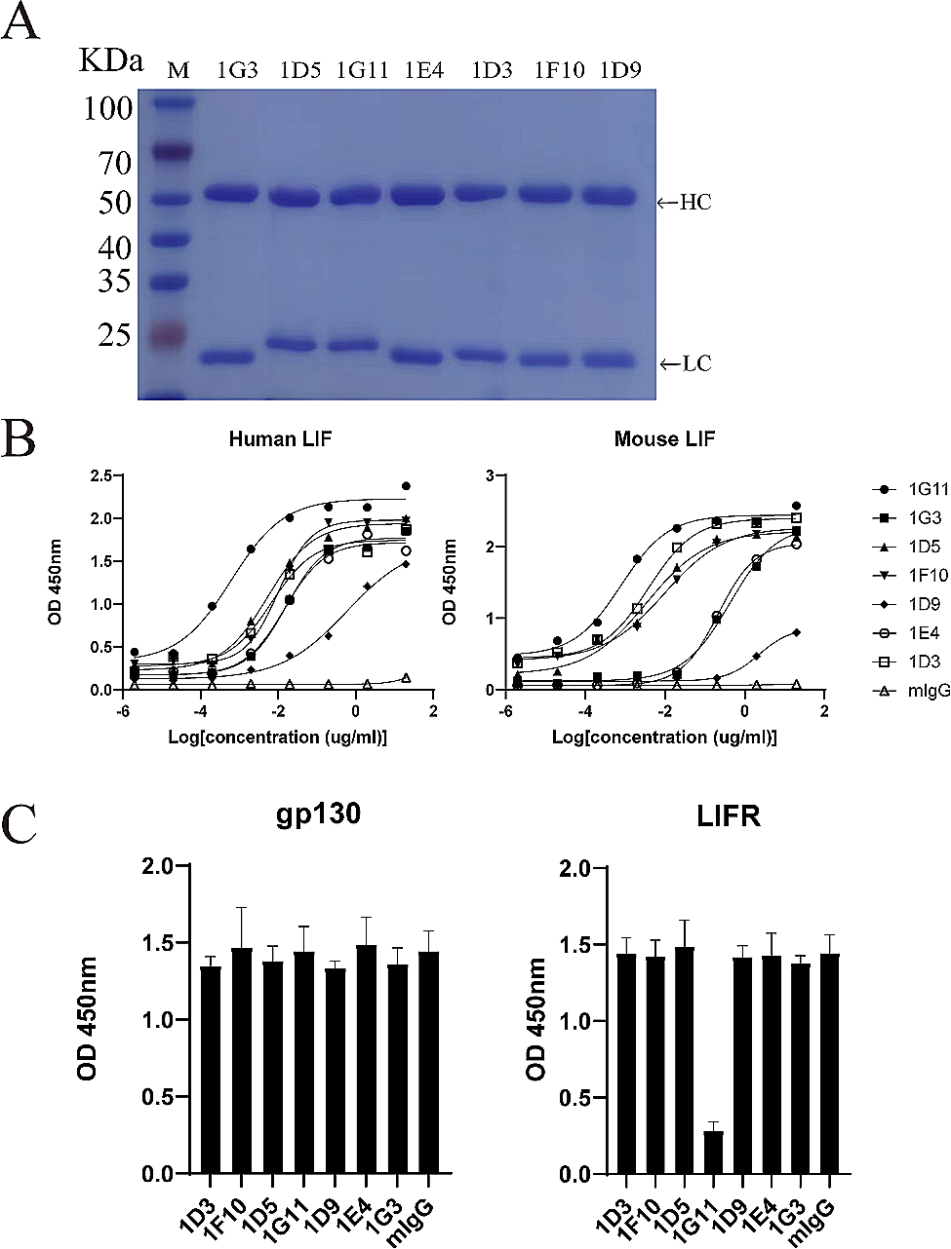
Expression and screening of recombinant monoclonal antibodies. (**A**) SDS-PAGE analysis of the purified antibodies under reducing conditions. M, molecular weight marker. (**B**) Dose dependent binding of antibodies to immobilized human LIF and mouse LIF, normal mouse IgG was used as a negative control. (**C**) Blocking activities of antibodies (2 µg/mL) were analyzed by competitive ELISA, *n* = 3

The 7 screened scFvs displayed on the phage surface were reformatted to complete mIgG2a antibodies. These sequences were synthesized and inserted into the expression plasmid pTT5. The plasmids were transfected into HEK293F cells. The cell transfection supernatants were centrifuged and purified by protein G affinity chromatography. SDS-PAGE analysis revealed that the purified products of these antibodies were composed heavy chain and light chain under reducing conditions, and the purities exceeded 95% (Fig. 2A).

To compare the binding activity of 7 antibodies to LIF, ELISA measurements were performed. All antibodies exhibited a dose-dependent binding activity to both hLIF and mLIF (Fig. 2B). The EC50 values are shown in Table 1. It is noteworthy that 1G11 has optimal binding activity.

**Table 1:** ELISA analysis of recombinant antibody binding to hLIF and mLIF

	EC50 (ng/ml)	
Clone	hLIF	mLIF
1G11	0.62	0.79
1G3	16.21	427.1
1D5	5.44	3.43
1F10	10.14	9.49
1D9	591.6	2307
1E4	14.65	215.5
1D3	6.69	3.82
mIgG	N/A	N/A

The binding activity of recombinant antibodies is expressed as EC50 values. N/A, not applicable

Competitive ELISA was performed to determine if these 7 antibodies could interfere with LIF binding to its receptors LIFR and gp130. Antibodies were incubated with hLIF-hFc and added to the microtiter plate wells coated with LIFR or gp130. HRP-conjugated goat anti-human IgG antibody was used to detect hLIF-hFc bound to LIFR or gp130. The results showed that 1G11 could significantly block LIF binding to LIFR, but did not affect LIF binding to gp130. None of the remaining 6 antibodies were observed to block LIF binding to either LIFR or gp130 (Fig. 2C). This finding was consistent with the functional activity of the antibodies. We selected 1G11 as a candidate antibody for further testing based on these results.

### The characterizations of 1G11 in vitro

**Fig. 3:**
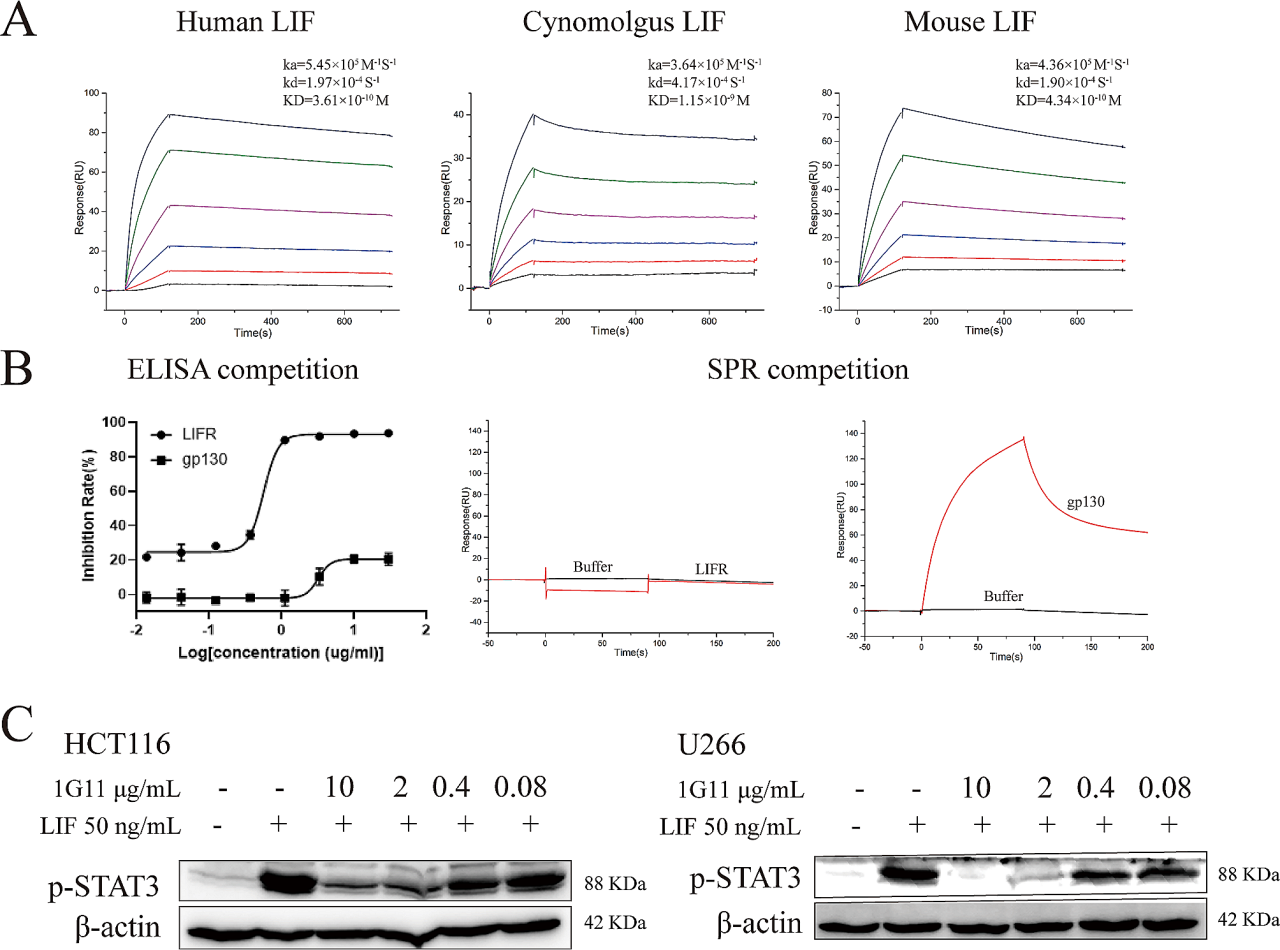
Characterization of antibody 1G11. (**A**) SPR affinity analysis of 1G11 binding to human, cynomolgus and mouse LIF. (**B**) Competitive ELISA and SPR analysis of 1G11. 1G11 competes with LIFR for binding to LIF, while 1G11 does not compete with gp130 for binding to LIF. (**C**) Western-bolt analysis for the inhibitory effect of 1G11 on LIF-induced p-STAT3

Multi-concentration SPR was utilized to evaluate the affinity of 1G11 to LIF. The results showed 1G11 not only recognized human LIF with excellent affinity (KD = 3.61 × 10^− 10^ M), but also recognized cynomolgus monkey LIF (KD = 1.15 × 10^− 9^ M) and mouse LIF (KD = 4.34 × 10^− 10^ M) (Fig. 3A). As measured by competitive ELISA, 1G11 showed strong competitive effects against LIFR, and weak competitive effects against gp130. 1G11 nearly completely blocked binding of LIF to LIFR at 3.3 ng/ml, with competitive inhibition rates more than 90% (Fig. 3B). However, competitive inhibition of gp130 by 1G11 was only 10% (Fig. 3B). Competitive SPR indicated that LIF could bind to both 1G11 and gp130 concurrently, but it could not bind to LIFR after binding to 1G11. These results demonstrated that 1G11 and LIFR share the same binding site on LIF, whereas 1G11 and gp130 have different binding sites on LIF.

Although our results showed that 1G11 can block LIF binding to its receptor (LIFR) at the protein level, we wondered whether 1G11 affects LIF binding to cells and blocks LIF function. We confirmed the functional activity of 1G11 on cells using western blots in vitro. We observed 1G11 dose-dependently inhibited LIF-induced p-STAT3 in U266 and HCT116 cells. The inhibition rates were greater than 90% at the 1G11 concentration of 2 µg/ml (Fig. 3C). Thus, the results suggested that 1G11 could inhibit the downstream STAT3 signaling pathway by blocking LIF binding to a single receptor (LIFR).

### Anti-tumor activity of 1G11 in vivo

**Fig. 4:**
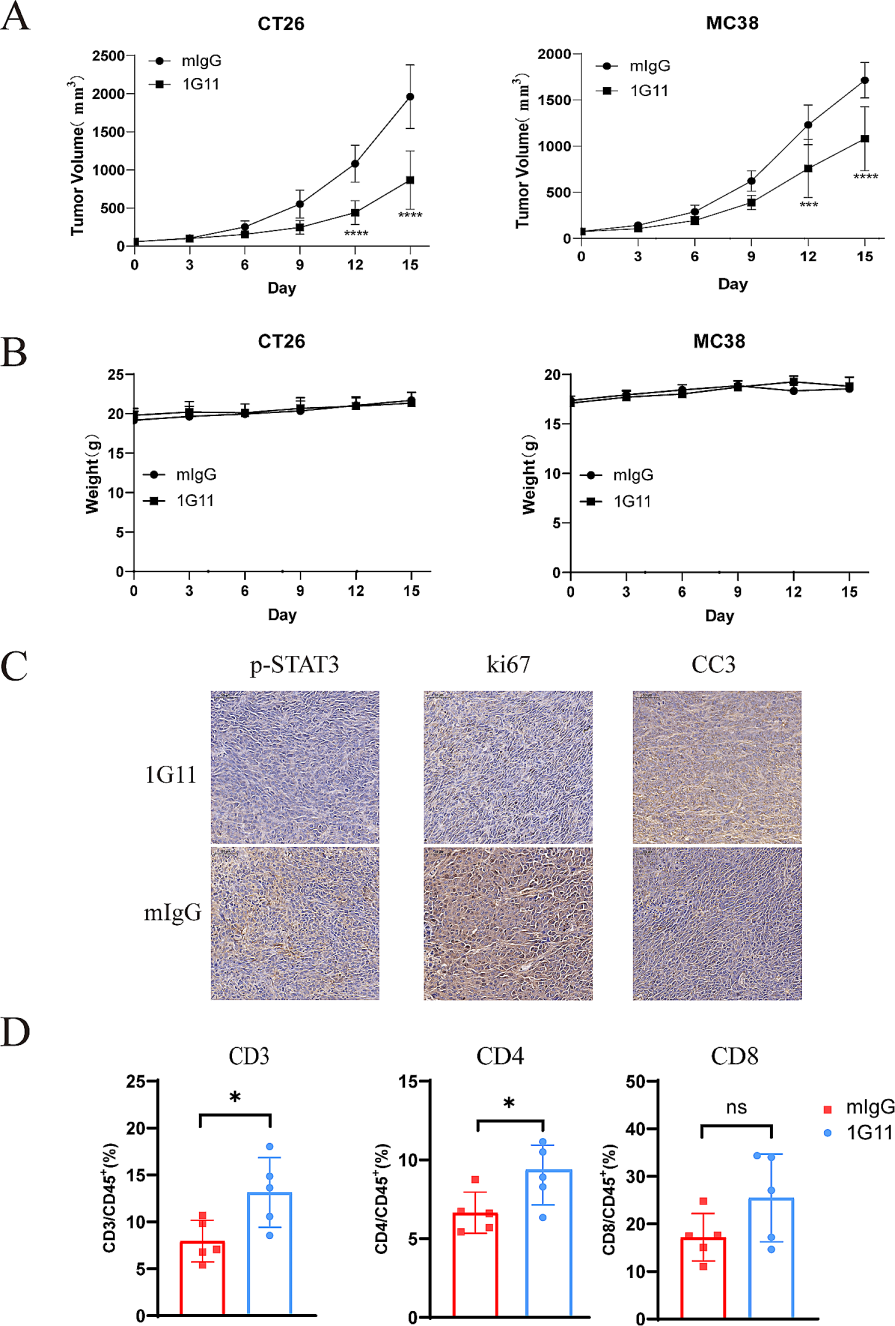
Antitumor effect of 1G11 in vivo. (**A**) Tumor growth curves of CT26 and MC38 mice. *n* = 5 per group. (**B**) Body weight monitoring of mice during the antibody treatment process. (**C**) IHC staining of p-STAT3, Ki67, and CC3 in tumor tissues (scale bar 50 μm). (**D**) Flow cytometry analysis of TME revealed the proportions of CD3 + T cells, CD4 + T cells and CD8 + T cells among the CD45 + cells. Data are mean ± SD. *, *P* < 0.05; **, *P* < 0.01; ***, *P* < 0.001; ****, *P* < 0.0001; ns, *P* ≥ 0.05

We established subcutaneous tumors of mouse CT26 and MC38 colorectal cancer cells to determine whether 1G11 inhibits tumor growth in vivo. In CT26 tumor-bearing mice, 1G11 significantly inhibited tumor growth. The TGI rate at the dose of 10 mg/kg was 55.8% (Fig. 4A). Similarly, 1G11 (10 mg/kg) also inhibited tumor growth in MC38 tumor-bearing mice and yielded a TGI rate of 36.9% (Fig. 4A). No body weight and behavior abnormalities were observed in mice during the treatment process (Fig. 4B).

At the end of treatment, we collected and fixed partially excised tumors and analyzed p-STAT3 protein expression by IHC staining. The 1G11 treatment group showed decreased p-STAT3 expression, which dramatically reduced the expression of the cell proliferation marker Ki67 (Fig. 4C). In contrast, the apoptosis-related marker CC3 exhibited a significant increase (Fig. 4C). The infiltrating immune cells in the tumor microenvironment (TME) of CT26 tumor-bearing mice were further examined. In the 1G11 group, the rate of CD8^+^/CD45^+^ in tumor tissues showed an increasing trend compared with the mIgG control, (Fig. 4D), and the rates of CD3^+^/CD45^+^ and CD4^+^/CD45^+^ increased significantly (Fig. 4D).

**Fig. 5:**
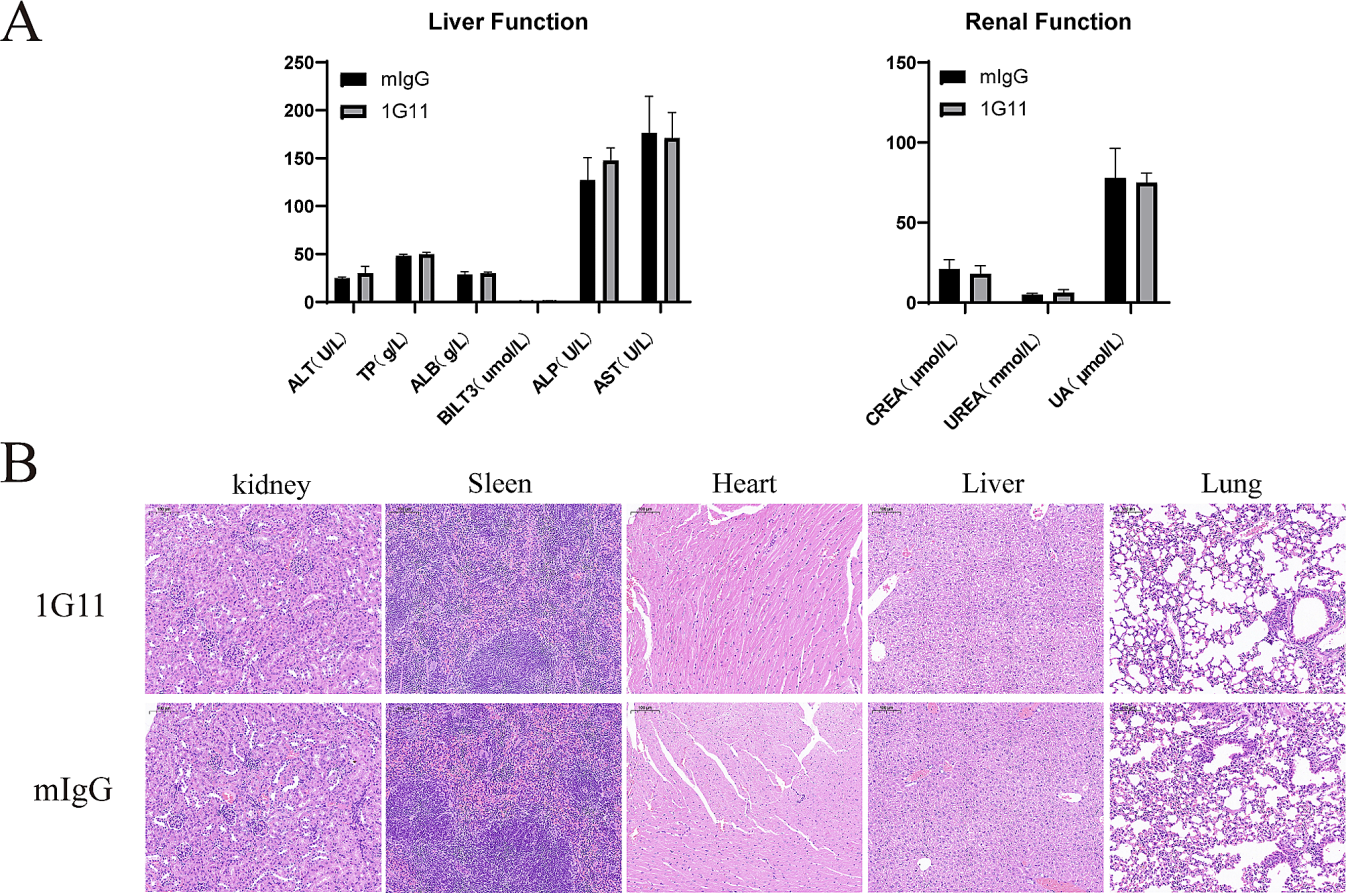
Preliminary safety evaluation of 1G11. (**A**) Blood biochemical indices of mice were determined after drug administration. (**B**) H&E staining of vital organs (scale bar 100 μm)

Additionally, we preliminarily evaluated the safety of 1G11. Biochemical analysis of blood samples and H&E staining of major organs showed no significant hepatorenal toxicity and tissue lesions were detected in the 10 mg/kg 1G11 group (Fig. 5A, B). Nevertheless, further investigations in monkeys are recommended to assess potential side effects prior to initiating clinical trials. Considering the effective tumor inhibition and excellent safety profile, 1G11 has potential as an anti-tumor drug.

## Discussion

The use of naive human scFv phage libraries has emerged as a prominent trend in antibody drug development. The amino acid sequences of these scFv antibodies derive from human entirely, which may reduce the risk of immunogenicity [18, 19]. Besides, the conversion of the scFv form of the antibody displayed on phage to the complete IgG form carries the risk of reduced affinity, which necessitates experimental validation [20, 21]. Our results showed that the expressed complete antibodies maintained a high affinity for LIF, consistent with the results of the phage supernatant assay.

Antibodies with cross-recognition activity across different species offer several advantages, particularly in the case of mice. These antibodies not only assist in pharmacological studies but also in the early detection of potential toxicity. Therefore, we investigated the binding of the phage supernatant to mouse LIF and identified antibodies with the desired affinity. Among them, the antibody 1G11 recognizes human, cynomolgus monkey, and mouse LIF with high affinity. This characteristic allows for the early investigation of the potential of 1G11.

In addition to affinity, the ability of antibodies to regulate signal transduction is crucial, particularly when targeting molecules with important functions. To evaluate the neutralizing capabilities of these antibodies at the protein level, we conducted competitive analyses. Our results showed that when LIF is bound to 1G11, it does not bind to LIFR but does bind to gp130. Upon binding of LIF, LIFR heterodimers with gp130. Subsequently, the C-terminal FNIII domain of receptors are then positioned precisely on the cell surface so that receptor-associated JAKs can be transphosphorylated [22, 23]. Thus, after 1G11 binds to LIF, LIFR is unable to bind to LIF and cannot forms a stable receptor complex with gp130. Consequently, conformational changes cannot take place and intracellular p-STAT3 signaling cannot be initiated. As we have observed that 1G11 significantly inhibited LIF-induced p-STAT3. Similarly, humanized anti-LIF antibody MSC-1 from AstraZeneca, which prevents LIF binding to gp130, also inhibits p-STAT3 [24].

The p-STAT3 protein is crucial for tumor cell growth [25]. The abnormal expression of LIF in colorectal cancer led to the rapid expansion of tumor cells by activating STAT3 signaling [12]. In our study, we found 1G11 blocked the LIF/LIFR/STAT3 axis in tumor cells in vitro. Consistent with our findings in vitro, 1G11 inhibited tumor growth in mice and reduced p-STAT3 expression in tumor tissues, as confirmed by IHC. Furthermore, we observed a decrease in Ki67 expression and an increase in CC3 expression. These results showed 1G11 can inhibit tumor cell growth and promote tumor cell apoptosis in vivo.

T cells are essential for anti-tumor immunity and increased T cell infiltration has the potential to improve positive prognostic indicators in various tumor types [26]. Several literatures have reported MSC-1 can modulate the effect of LIF on chemokine secretion in macrophages and improve CD8^+^ T cell infiltration [27]. Surprisingly, we found that 1G11 also has the effect of modulating immune cells. As the flow cytometry result of 1G11 increases CD3^+^, CD4^+^ and CD8^+^ T cell infiltration in tumor tissue, it may be able to act synergistically with anti-PD1 antibodies or other immune checkpoint inhibitors. Our data suggest that the anti-tumor effect of 1G11 is multifaceted and the specific mechanisms by which 1G11 affects immune cells deserve further investigation.

## Conclusions

In this work, an antagonist antibody targeting LIF has been isolated and characterized from a naive human scFv phage library. The anti-LIF antibody 1G11 exhibits excellent affinity with human, cynomolgus monkey and mouse LIF and competitively blocks LIF binding to LIFR. Furthermore, 1G11 inhibited LIF-induced p-STAT3 in vitro, and reduced p-STAT3 levels in tumor cells in tumor-bearing mice, thereby inhibiting tumor growth. FACS analysis of tumor tissue revealed that 1G11 administration increased immune cell infiltration. Collectively, our results highlight the potential of the LIF/LIFR antagonist antibody 1G11 as an effective tumor therapeutic strategy.

### Electronic supplementary material

Below is the link to the electronic supplementary material.

Supplementary file 1: Original western blots were included for Fig. 3C

## Data Availability

The datasets presented in the study are included in the article/supplementary file. Further inquiries can be directed to the corresponding authors.
