# Supplementary material for: Antagonist anti-LIF antibody derived from naive human scFv phage library inhibited tumor growth in mice
Source: BMC Immunol. 2024 Aug 22;25:56. doi: 10.1186/s12865-024-00636-w (PMC11340043; doi:10.1186/s12865-024-00636-w)
Supplement: Supplementary file 1 — Original western blots were included for Fig. 3C [file 12865_2024_636_MOESM1_ESM.pptx]

## Slide 1
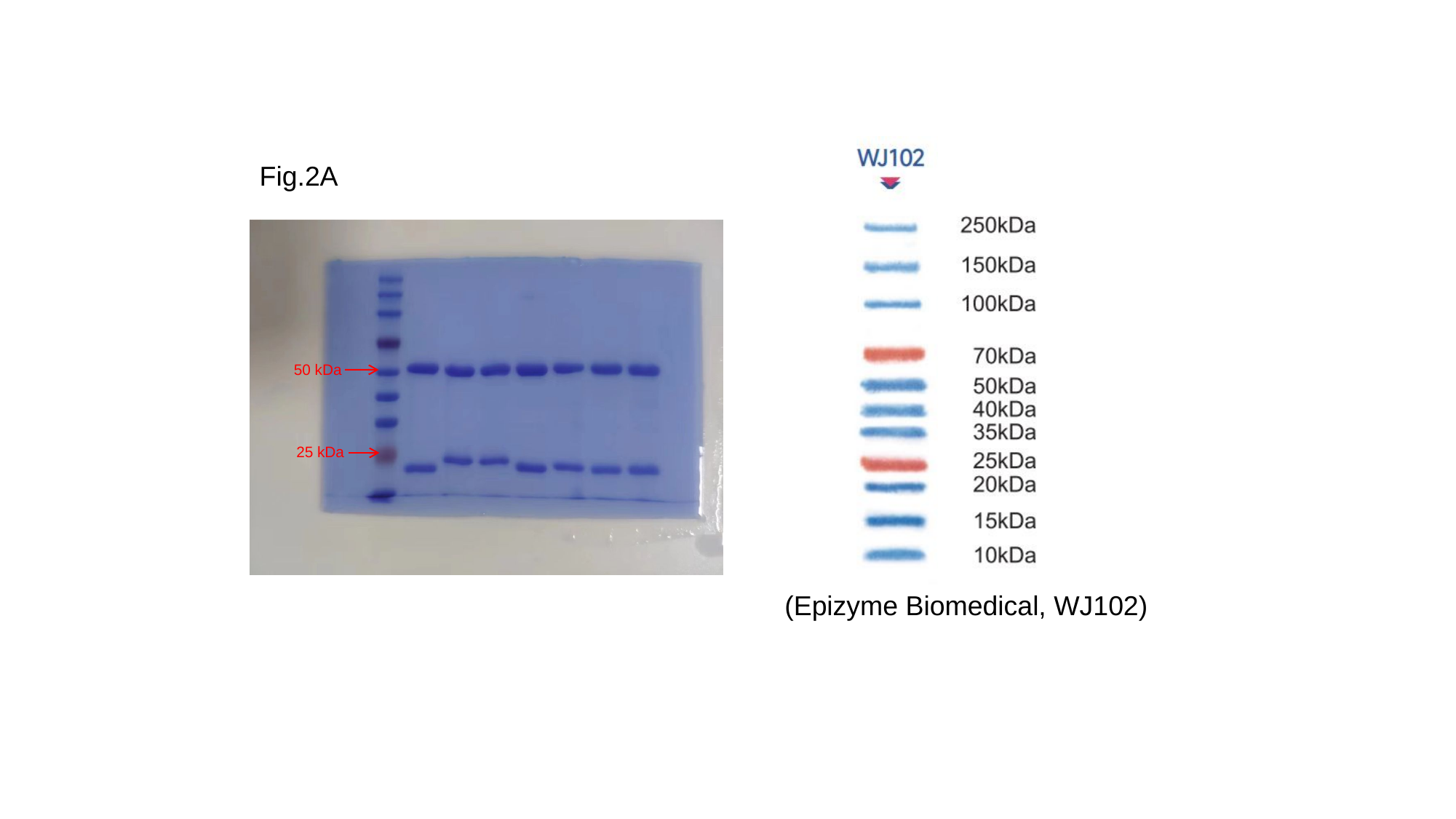

Fig.2A
50 kDa
25 kDa
(Epizyme Biomedical, WJ102)

## Slide 2
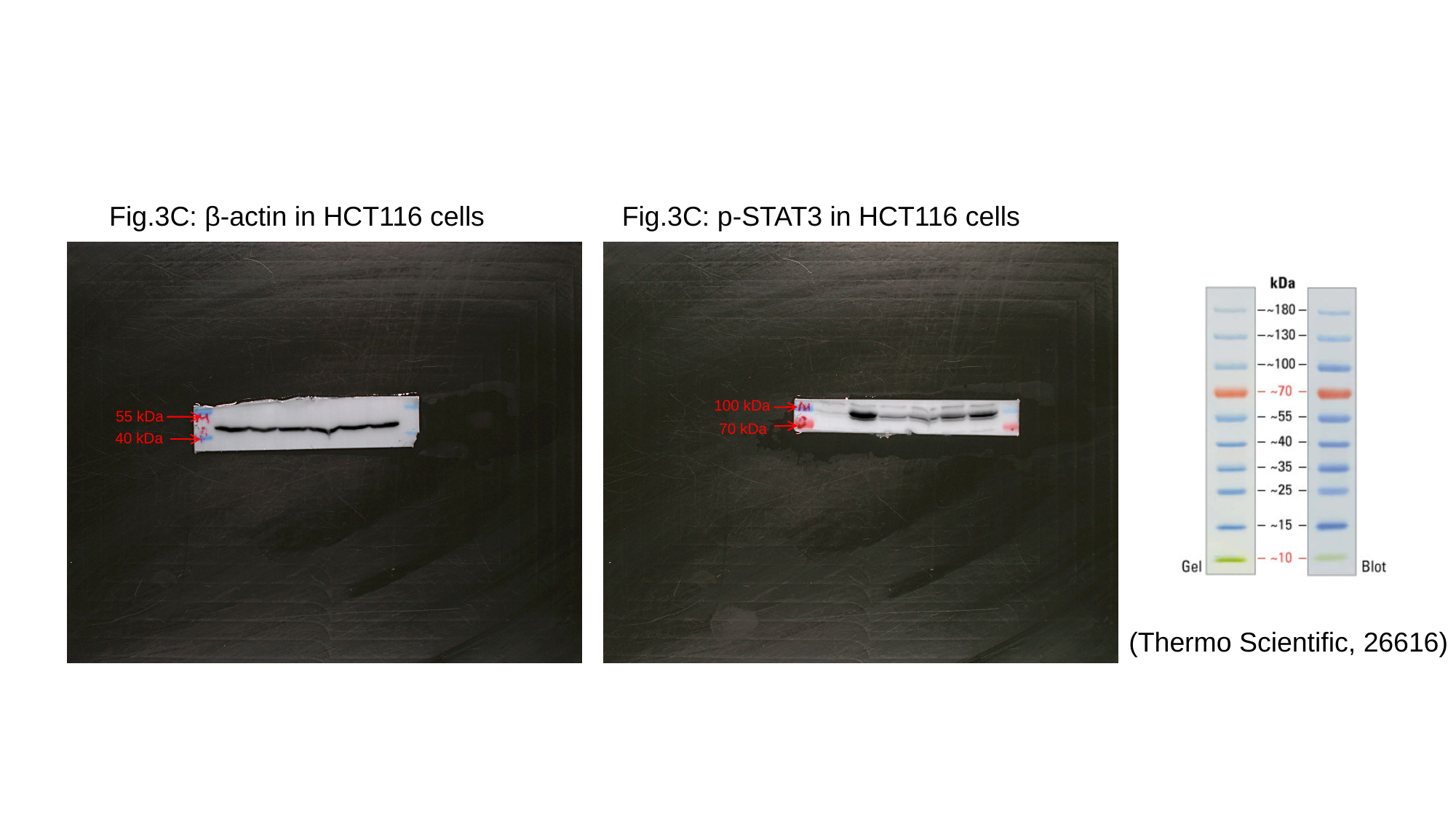

Fig.3C: β-actin in HCT116 cells
 Fig.3C: p-STAT3 in HCT116 cells
100 kDa
55 kDa
70 kDa
40 kDa
(Thermo Scientific, 26616)

## Slide 3
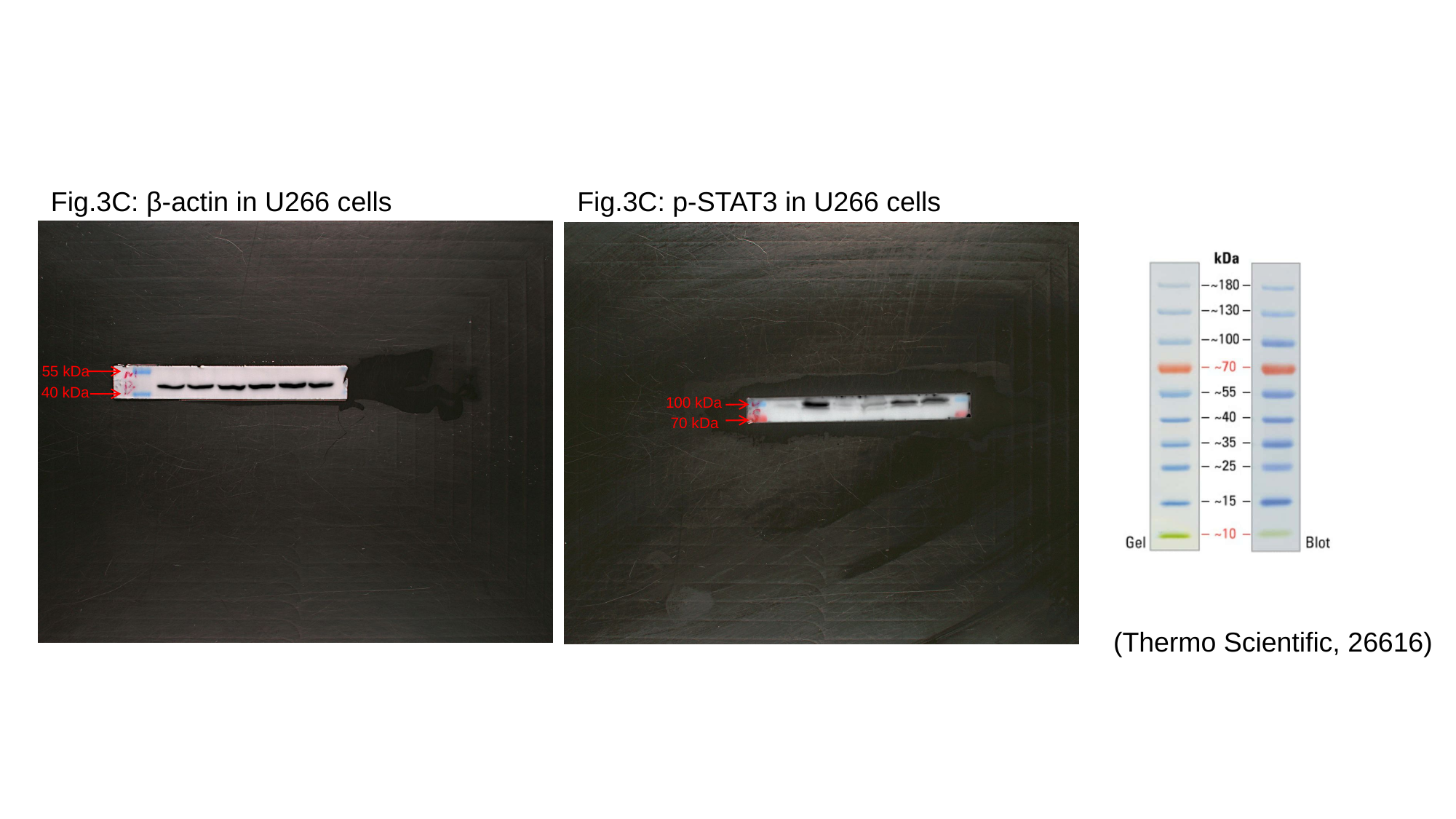

Fig.3C: β-actin in U266 cells
Fig.3C: p-STAT3 in U266 cells
55 kDa
40 kDa
100 kDa
70 kDa
(Thermo Scientific, 26616)
